# The Transformative Role of Artificial Intelligence in Dentistry: A Comprehensive Overview Part 2: The Promise and Perils, and the International Dental Federation Communique

**DOI:** 10.1016/j.identj.2025.02.006

**Published:** 2025-02-25

**Authors:** Nozimjon Tuygunov, Lakshman Samaranayake, Zohaib Khurshid, Paak Rewthamrongsris, Falk Schwendicke, Thanaphum Osathanon, Noor Azlin Yahya

**Affiliations:** a Faculty of Dentistry, Kimyo International University in Tashkent, Tashkent, Uzbekistan; b Faculty of Dentistry, University of Hong Kong, Sai Ying Pun, Hong Kong; c Dr DY Patil Dental College and Hospital, Dr DY Patil Vidyapeeth, Pimpri, Pune, India; d Center of Excellence for Dental Stem Cell Biology, Faculty of Dentistry, Chulalongkorn University, Bangkok, Thailand; e Department of Prosthodontics and Dental Implantology, College of Dentistry, King Faisal University, Al-Ahsa, Saudi Arabia; f Department of Anatomy, Faculty of Dentistry, Center of Excellence for Regenerative Dentistry, Chulalongkorn University, Bangkok, Thailand; g Clinic for Conservative Dentistry and Periodontology, University Hospital of the Ludwig-Maximilians- University Munich, Munich, Germany; h Department of Restorative Dentistry, Faculty of Dentistry, Universiti Malaya, Kuala Lumpur, Malaysia

**Keywords:** Artificial Intelligence (AI), Machine Learning, Patient Communication, Ethics, AI Integration

## Abstract

In the final part of this two part article on artificial intelligence (AI) in dentistry we review its transformative role, focusing on AI in dental education, patient communications, challenges of integration, strategies to overcome barriers, ethical considerations, and finally, the recently released International Dental Federation (FDI) Communique (white paper) on AI in Dentistry. AI in dental education is highlighted for its potential in enhancing theoretical and practical dimensions, including patient telemonitoring and virtual training ecosystems. Challenges of AI integration in dentistry are outlined, such as data availability, bias, and human accountability. Strategies to overcome these challenges include promoting AI literacy, establishing regulations, and focusing on specific AI implementations. Ethical considerations in AI integration within dentistry, such as patient privacy and algorithm bias, are emphasized. The need for clear guidelines and ongoing evaluation of AI systems is crucial. The FDI White Paper on AI in Dentistry provides insights into the significance of AI in oral care, dental education, and research, along with standards for governance. It discusses AI's impact on individual patients, community health, dental education, and research. The paper addresses biases, limited generalizability, accessibility, and regulatory requirements for AI in dental practice. In conclusion, AI plays a significant role in modern dental care, offering benefits in diagnosis, treatment planning, and decision-making. While facing challenges, strategic initiatives focusing on AI literacy, regulations, and targeted implementations can help overcome barriers and maximize the potential of AI in dentistry. Ethical considerations and ongoing evaluation are essential for ensuring responsible, effective and efficacious deployment of AI technologies in dental ecosystem.

## Introduction

In Part 1 of this article we reviewed the fundamentals of AI and the current applications of AI in clinical dental practice. Here we review the use of AI in dental education, patient communications and the challenges of AI integration in Dentistry. We further suggest some strategies and initiatives to overcome barriers to AI deployment as well as ethical considerations related to AI in dentistry. The review concludes with a succinct summary of the recent FDI Policy Statement on AI in dentistry.

### AI in dental education

AI is promising for enhancing dental education across theoretical and practical dimensions. In theoretical education, AI aids in the analysis of patient data, formulation of treatment plans, and simulation of clinical scenarios. On the pedagogic front, AI facilitates patient telemonitoring, creates virtual training environments, and improves student evaluation and patient management.^1^ The rapid advancements in AI technology make it essential for dental practitioners to acquire the knowledge and skills needed to effectively integrate AI into dental treatments.^2^ The following briefly describes the transformative potential of AI in dental education, focusing on two pivotal domains: (1) *Theoretical Education,* which emphasises the development of soft skills, research methodologies, and publication practices and (2) *Practical/Clinical Education*, which highlights advancements in direct patient care training and clinical skill enhancement.

Both theoretical and practical realms within dental education are undergoing significant transformations in their traditional workflows. In theoretical education, traditional diagnostic theories are encountering algorithms capable of analysing patient data and making diagnoses without the need for explicit understanding by clinicians. Digital communication tools are enabling personalised interactions, while semi-automated responses to frequently asked questions can mimic the style and tone of healthcare professionals. Moreover, advanced Large Language Model (LLMs), capable of generating unique texts, are poised to disrupt conventional approaches to essay writing and research publication.^3^

In practical education, AI-powered telemonitoring approaches are adapted for patient care across all stages of treatment: before, during, and after. This approach has the potential to transform dental care. With the Internet of Things devices, ie, smart devices, health data can be recorded and analysed by AI to further inform, instruct, or respond to patient conditions. Dental professionals can design the objectives of AI interventions in practical/clinical education. AI-assisted systems improve treatment skills for inexperienced dental students in various procedures, for example, dental implant placement.^4^

This shift in practical training will be accompanied by AI-generated realistic virtual environments for training purposes. Moreover, AI will enhance student evaluation methods and contribute to improved patient care, with its role extending beyond personalised therapy to encompass various aspects of patient management.^5^ LLMs effectively handle comprehension questions,^6^ implicating its use in patient communication components in education. Notably, more patient interaction with AI chatbots may compromise human empathy.^3^ Hence, implementing AI in clinical training should be carefully harmonised between technology-assisted learning experience and the human touch in patient care.

There are numerous avenues for integrating AI into the dental education curriculum. One approach is utilising AI-powered simulation. This supports diagnosis and treatment stages to enhance the student learning experience. Additionally, AI-generated information could assist in clinical decision-making by providing possible prediction, prognosis, and treatment options. Another method involves employing AI-powered image analysis and interpretation to enhance student comprehension of radiographic images. Furthermore, AI can potentially improve operational effectiveness by automating administrative tasks, including patient appointments and dental record management.

In terms of publishing and manuscript creation, AI could greatly benefit researchers in academia,^3^ where the rapid dissemination of research findings is crucial. The traditional practice of outsourcing poorly written drafts to paid academic writers in the industry can now be efficiently handled by tools like ChatGPT, Deepseek and similar applications, which are freely accessible to researchers. This advancement is particularly advantageous for countries with limited resources, especially where English is not the native language. LLMs can also be used as co-pilots to generate new ideas for research in dentistry.^31^ Moreover, these large language models can potentially enhance the peer review process, thereby elevating the overall quality of scientific publications.^3^ Several publishers now allow the controlled use of AI for drafting manuscripts and enhancing their grammatical and syntactical accuracy. While these LLMs, such as ChatGPT, offer immense benefits to researchers, there is a risk of misuse, especially in the context of generating review papers. Furthermore, these models may sometimes incorrectly contextualise information and generate entirely fabricated references, a phenomenon referred to as "hallucination". These examples illustrate a fraction of the possibilities for incorporating AI into dental education. Ultimately, the specific implementation of AI will depend on the unique objectives and requirements of each educational institution.^7^^,^^8^

### AI for patient communication

As mentioned above, AI can be integrated into numerous patient communications for educational and consultation purposes. Generative AI with the LLM models has been extensively investigated as the information provider in various scenarios in dentistry. For instance, chatbot (based on generative AI and LLM) consultations could provide some basic information for patients.^9^ One study has compared the effectiveness of an embedded GPT model and ChatGPT-3.5 turbo in delivering post-operative dental care advice. Results indicate that the embedded GPT model outperformed ChatGPT in accuracy (62.5% vs 52.5%) and clarity (72.5% vs 67.5%). However, both models exhibited similar performance regarding relevance and contemporaneous knowledge. A similar study shows that different LLMs demonstrated variations in accuracy in response to periodontal and endodontics-related healthcare questions, in a self-evaluation study by patients.^10^ ChatGPT was used for questions and consultations in Oral and Maxillofacial Surgery with 71.7% accuracy.^11^ In contrast, ChatGPT responses exhibited only 25.6% accuracy in the field of removable and tooth-supported fixed dental prostheses.^12^ In Orthodontics, ChatGPT responded to questions regarding clear aligner treatment with 58% accuracy.^13^ It should be noted that LLMs demonstrate a superior performance in English compared with other languages (i.e, Chinese).^10^ These results show the promise of LLMs across a wide range of dental fields for patient communication. However, the low accuracy in some areas necessitates caution in using and trusting LLMs-generated answers.^9^

### Challenges related to AI integration in dentistry

Numerous challenges confront the integration of AI into dental practice. These included data availability, generalizability, explainability, and reproducibility, and these exist in both image and speech processing realms. Some of such challenges, particularly in model evaluation, have been previously outlined^14^, ^15^, ^16^, ^17^, ^18^ and we review these in brief, as follows.

### Data availability, accuracy, privacy, and security

The availability of data, along with concerns regarding data privacy and security in AI development for healthcare, presents both substantial challenges and significant opportunities. The demand for high-quality training datasets is apparent; however, the limitations imposed by local data storage and the inherently sensitive character of clinical text data complicate the processes of access and utilisation. Additionally, incomplete or inaccurate records affect the performance of AI training. Consequently, training and testing datasets for dental AI are often limited in their size. The facilitation of data exchange and federated learning will necessitate the collaboration of broader coalitions of dental researchers.^19^^,^^20^ Moreover, data exchange also implies the usage of standardised terminology (eg, SNOMED CT, SNODENT), clinical procedures, and data models (e.g. Fast Healthcare Interoperability Resources) in order to effectively share data.^14^ Proposed interoperable electronic hospital record systems aim to improve patient safety and streamline healthcare delivery.^21^ Additionally, the ethical obligation to safeguard patient information accentuates the critical need for robust security protocols to mitigate potential cyber threats.^22^ Data de-identification is often required to navigate the complexities of privacy legislation. It is imperative to balance preserving patient confidentiality and ensuring the precision and efficacy of AI models.^23^, ^24^, ^25^ Continuous progress in privacy-preserving methodologies is vital for cultivating a secure framework for AI applications within healthcare use. In USA the NIH Common Fund's Bridge to Artificial Intelligence (Bridge2AI) initiative has been established to facilitate the responsible application of AI, aiming to deliver data sets that are ethically obtained, reliable, and readily accessible by integrating diverse sectors, which encompass healthcare professionals and technical specialists alongside social scientists and humanists.^26^ Ultimately, a unified initiative among researchers, dental practitioners, and regulatory authorities is crucial for addressing these challenges and maximising the potential of AI technologies while protecting patient rights and data integrity.

### Bias, fairness, and generalizability

As mentioned above, the quality and quantity of input data are crucial for AI efficacy^27^ (`Rubbish in, rubbish out`). Bias such as gender, socioeconomic background, and ethnicity can subsequently result in inferior performance for specific demographic populations, ultimately impacting the quality of healthcare services.^28^^,^^29^ Additionally, AI models trained by one dataset may not generalise well when applied to other datasets. In one real-world COVID-19 screening staudy, notable discrepancies in performance emerged between high-income (USA) and low- to middle-income countries (Vietnam), primarily attributed to data imbalances, highlighting the issue of bias and fairness in AI models.^30^ However, the application of algorithmic bias mitigation techniques markedly enhances the equity of AI models while preserving superior diagnostic sensitivity.^30^ Studies in AI pertaining to orthodontics suggest that biases emerge due to the influence of subject matter content experts, inadequate diversity within training datasets, and a lack of sufficient representation of racial and ethnic minority groups. When models derived from the general population are applied to high-risk or underrepresented cohorts, this results in errors characterised by "distribution-shift".^31^ All stakeholders must address biases throughout the model-developing process by ensuring diverse team composition and utilising inclusive datasets; creating race-specific datasets and addressing data gaps can alleviate representation bias while prioritising patient-centric outcomes and involving marginalised communities in research fosters equity. Furthermore, establishing clear data standards, thorough reporting, and external validation of such models is critical for achieving reliable, equitable, and reproducible clinical outcomes.^31^

### Human accountability

AI provides numerous benefits for dental practitioners. It can assist in multifaced tasks, including diagnosis, treatment planning, clinical decision-making, prediction and prognosis. However, one must still emphasise the role of humans in these processes. Human expertise remains irreplaceable, encompassing empathy, ethical considerations, and the ability to interpret complex information that AI may not completely comprehend. Numerous AI models are frequently regarded as black box systems, obstructing human comprehension of the mechanisms involved in output generation.^26^ Explainable Artificial Intelligence (XAI) is a domain that is progressing rapidly so as to mitigate concerns associated with opaque algorithms, underscoring the initiatives to enhance comprehensibility, transparency, and reliability of AI systems.^26^^,^^32^ The achievement of human accountability depends upon the individual's understanding of the foundational rationale that informs the AI's outputs or their decision-making processes, a concept referred to as Explainability. Transparency is particularly vital in using AI in healthcare, where understanding the rationale behind decisions can significantly impact individuals. Ultimately, advancing our comprehension of AI's inner workings not only promotes accountability but also empowers users to engage more critically with technology that increasingly shapes their realities.

### Rapid development of AI for real-world utilisation

AI integration into healthcare faces significant delays in transitioning from research to production due to rigorous testing, industry standards, and system integration requirements, ensuring safety and privacy of patients. Developing AI models involves time- and labour-consuming processes. For example, creating predictive models for diseases or automated diagnostic tools requires extensive, labelled datasets by experts, computational resources, and refinement, that are likely to hinder timely advancements in patient care. This challenge underscores the need for synergistic partnerships and collaboration among researchers, dental practitioners, and technological enterprises to optimise methodologies and expedite AI deployment.

### Strategies and initiatives to overcome barriers in AI deployment

Considering the challenges outlined above, various recommendations have been proposed to facilitate the effective adoption and widespread deployment of AI-based tools across healthcare organisations^18^:

### AI in healthcare innovation

AI is bound to disrupt multiple processes and workflows in dentistry. In some situations, it can potentially be viewed as a threat when utilised inappropriately. It is crucial to develop the perception of AI as a facilitator. AI should be conceptualised as an instrument for the automation of routine tasks and the reduction of workload, facilitating dental professionals to focus on dental care and service. Guidance and training are required to foster appropriate AI use. The risk management for AI autonomous capabilities should be addressed and monitored to assure patient safety and privacy.^26^ The systematic evaluation of AI algorithms and their results across diverse patient demographics with integrating ethical considerations is crucial to address bias, fairness, and generalisation. Additionally, user feedback can be implemented to enhance the optimisation of AI in dental healthcare. By implementing these strategies, dental healthcare organisations can foster a collaborative environment where AI tools and human workers synergistically enhance patient care and outcomes.^33^^,^^34^

*AI literacy enhancement:* As integrating AI in dental healthcare increases, promoting AI literacy for current and future dental professionals is essential.^35^ Dental practitioners must be equipped with knowledge and skills for critically evaluating and utilising AI effectively and ethically. AI education must be incorporated into the dental curricula, of not only the budding dental practitioners but also for the auxiliary practitioners of dental care. University academics must also play a key role in enhancing student understanding of AI in dental healthcare. AI algorithm development, healthcare data analysis, and ethical considerations, with hands-on projects to apply knowledge in real-world scenarios should be integrated into acdemia. Ethics modules addressing algorithmic bias, data privacy, and human accountability to ensure that dental professionals are skilled in AI and aware of their ethical responsibilities should also be developed and implemented.

### AI regulatory frameworks

These play a crucial role in ensuring the safe and ethical deployment of AI in healthcare.^36^ Developing robust regulation for AI in healthcare accelerates adoption by instilling confidence in the technology among stakeholders. Regulation must focus on the transparency of AI algorithms to validate reliability and fairness. In addition, quality control porcesses must be in place to ensure patient safety, alongside the establishment of liability frameworks that should be concerned to address risks from incorrect interpretation and recommendations related to AI. FInally, ensuring ethical AI in healthcare requires rigorous ethical principles to prevent bias, robust data privacy protections to comply with available data protection regulations, stringent security protocols to guard against cyber threats, and dynamic monitoring to maintain compliance with evolving legal and ethical standards.^37^

*Specific focus on AI implementation:* To successfully integrate AI into dental healthcare, organisations should pinpoint specific challenges AI can solve and ensure its implementation to align with current workflows. This allows the effective deployment of AI and to cultivate the perception of AI as an assistant in the organisation. For example, AI can be set to a preliminary screening of radiographs to provide a summary for interpretation prior to being confirmed by oral radiologists. This could facilitate the workflow and reduce the workload of dentists. The implementation necessitates systematic observation and assessment to ascertain their proper application and effectiveness, and thereby determine whether to continue or cease its utilisation. Significantly, this approach pertaining to the targeted AI application facilitates the fiscal management of the organisation to ensure a sustained dedication to the implementation of AI. Such investment transcends the preliminary procurement of AI systems and includes recurrent expenditures, for maintenance, security, licensing, and infrastructure.

A summary of the primary challenges of integrating AI in Dentistry and strategies to overcome them are shown in the Table.

**Table:** Challenges to AI integration into dentistry, and strategies to overcome these barriers.

Challenges to AI integration	Overcoming strategies
*Data availability, accuracy, privacy, and security*	Collaborate in data sharing; Explore federated learning; Reach consensus on data exchange standards; Establish standardized terminology; Implement interoperable electronic hospital record systems; Address data fragmentation;
*Bias, fairness, and generalizability*	Curate high-quality data; Implement proactive auditing of algorithms; Ensure diverse representation in datasets
*Generalizability*	Establish standardized terminology; Ensure diverse representation in datasets
*Human accountability*	Shift perception of AI; Align human and AI incentives; Develop explanation techniques; Ensure transparency and oversight
*Rapid development of AI for real-world utilisation*	Streamline development lifecycle; Implement tools for faster processes
*AI in healthcare innovation*	Highlight its role in task automation; Emphasize human responsibility; Implement reproducibility standards; Explore deterministic approaches; Develop clinically relevant metrics; Implement robust security measures; Ensure compliance with regulations
*AI literacy enhancement*	Incorporate AI education; Offer life long learning opportunities; Introduce guidance and training; Foster transparency and teamwork
*AI regulation implementation*	Develop comprehensive frameworks; Address transparency and ethics
*Specific focus on AI implementation*	Identify specific problems; Align implementation with care goals; Develop cost-effective labelling methods; Prioritize sustained commitment; Allocate resources for maintenance

By implementing these initiatives, NGOs, universities and other health care organisations can ensure that future healthcare professionals are well-prepared to harness the potential of AI for improving patient care and driving innovation in healthcare delivery.

### Ethical considerations related to AI in dentistry

Ethical considerations surrounding the integration of AI into dentistry are increasingly subjected to scrutiny and debate. One primary ethical concern revolves around patient privacy and data security. AI systems in dentistry often rely on vast amounts of patient data for training and analysis. Dentists and healthcare organisations must ensure that patient information is handled securely. In compliance with local, regional and federal data protection regulations to prevent unauthorized access and protect patient confidentiality.

Another critical ethical consideration is the issue of `algorithm bias`. AI algorithms can inadvertently perpetuate biases present in the data used to train them, leading to discriminatory outcomes. It is essential for dental professionals to carefully evaluate AI algorithms to mitigate bias and ensure fair and equitable treatment for all patients.

Furthermore, the impact of AI on the doctor-patient relationship is a significant ethical concern. As AI technologies become more integrated into dental practices, there is a risk of dehumanizing patient care and diminishing the importance of human interaction in healthcare. Dentists must strike a balance between leveraging AI for improved diagnostic accuracy and treatment planning while maintaining the essential human touch in patient care.

To address these ethical challenges, dental professionals and policymakers must establish clear guidelines and regulations governing the use of AI in dentistry, some of which are addressed in the recently produced FDI white paper on AI in dentistry. As AI technologies mature further in the fullness of time, frameworks should be developed to ensure transparency, accountability, and fairness in the deployment of AI in our clinical profession. Additionally, ongoing monitoring and evaluation of AI systems are crucial to identify and address potential ethical issues that may arise during their use.^38^

## FDI white paper on AI

The White Paper on “Artificial Intelligence for Dentistry” written by FDI Artificial Intelligence Working Group^39^ provides a concise review of the significance of AI in dentistry. The transformation of oral care for both individuals and the community, dental education, and dental research are described in the white paper. Additionally, the standards for good governance in the use of AI in dentistry are also described in alignment with FDI's Vision 2030. An outline of theFDI communique is provided below.

Focusing on individual patients, AI is applied to image analysis, data synthesis, prediction, treatment planning, and patient interaction. AI-based image analysis, including radiographs, intraoral scans, photographs, and near-infrared transillumination images, demonstrates high accuracy, and in some studies, the accuracy is higher than those evaluated by inexperienced dental practitioners. However, dental professionals must critically evaluate the AI products in this area prior to implementing in patient treatment. AI in data synthesis and prediction leverage the patients’ data to understand their risks and needs, allowing the efficiency and specificity of oral care, so-called P4 dentistry: personalised, precise, preventive and participatory dentistry. Despite the acceptable identification of risk factors, the predictive ability of AI remains limited. With AI tools, the “one-stop” dental care can transform into interactive and engaging life-long interactions with patients, such as daily behaviour monitoring in personalised preventive programs. AI can also support virtual communication and monitoring, enhancing patient engagement and interaction.

AI's roles in community and public health are also explored in the white paper. AI can be used in remote synchronous and asynchronous communication, increasing affordability, accessibility and equity of care. AI can also leverage big data derived from public health databases to predict healthcare demands and to develop workforce and public health service plans and policies.

AI has a significant impact on dental education and workforce development. AI analyses of multiple variables derived from both historical and contemporaneous datasets could be used to forecast future workforce needs and demands for efficient workforce planning. AI and augmented and virtual reality-based applications should be increasingly introduced into dental curricula as AI literacy is bound to be a challenge in dental education. The critical evaluation of choosing AI tools and their particular applications is crucial in this context. In addition, knowledge of data science is another essential component of AI evaluation.

In terms of dental research, AI tools that are currently available are able to handle big, complex datasets, allowing the identification of new, hitherto unimagined perspectives. However, with the current limitations of data quality, AI in oral and craniofacial research will be a challenging prospect. Reporting standards on AI in dental research should be adhered to. Lastly, the dental research community should be equipped with digital literacy for the appraisal of AI research and development.

Bias, limited generalizability, accessibility, interoperability, truthfulness, and implementation and maintenance regarding the use of AI (outilned in detialed in this article) are also discussed in the FDI whte paper. For instance, as AI datasets have been usually generated from those in more affluent and healthier populations, biases that disadvantage underrepresented groups are inevitable. The interoperability of data systems remains a significant challenge. The format and structure of data, as well as the employed terminology are the significant aspects to be considered in this context. Data interoperability in dentistry should be urgently discussed, aiming at an agreed standard for data structure and terminology. Accurate data labelling is critical for AI development, with annotator expertise significantly influencing quality. Disagreements among experts in regards to the above crtical issues should be managed through consensus and hierarchical reviews.

Further, strategies to ensure safety, accuracy, and efficacy of AI use in dental practice should be regulated, documented, widely disseminated and implemented . For this purpose guidance could be derived from WHO consensus promulgations with six primary principles on AI focused on the implementation of governance strategies, viz; i) to protect human autonomy, ii) to ensure responsible use of AI, iii) to develop AI that is transparent, explainable and intelligible, iv) to foster human responsibility and accountability, v) to ensure inclusiveness and equity, and vi) to promote responsive and sustainable AI. Besides, the governance strategies of the EU Commission, the USA Center of Disease Control and Prevention, and the USA Food and Drug Administration are discussed in the white paper.

Several calls for action are listed. The dental community should focus on the improvement of data access for AI training with the concern of data protection and ethical issues. The use of AI should be promoted along with critical appraisal. Active research must be strengthened to demonstrate the evidence of efficacy and use cases. AI literary should be enhanced by implementing AI education into the curriculum for both graduate and postgraduate training.

## Conclusions

AI has become an exceptionally prominent feature of contemporary dental care with profound ramifications. The integration of AI-based technologies has shown great potential in various aspects of the dental practice, from diagnosis and treatment planning to clinical decision-making and prognosis prediction in numerous aspects of dental speciality. It benefits both individual and community levels of care. The implementation of generative AI has recently been introduced as a healthcare tool for dental practitioners. There are several challenges to AI integration in dentistry, ranging from technological aspects, AI literacy, and governance of AI use. AI integration also faces considerable obstacles, such as limited data availability, bias, and data privacy concerns. To overcome these barriers, strategic initiatives are needed, including cultivating new perspectives on AI, establishing clear objectives, fostering a supportive work culture, prudent investments and regulatory frameworks, and enhancing AI literacy.

## Conflict of interest

None disclosed.
